# MiR-138 Acts as a Tumor Suppressor by Targeting *EZH2* and Enhances Cisplatin-Induced Apoptosis in Osteosarcoma Cells

**DOI:** 10.1371/journal.pone.0150026

**Published:** 2016-03-28

**Authors:** Ziqiang Zhu, Jinshan Tang, Jianqiang Wang, Gang Duan, Lei Zhou, Xiaoqing Zhou

**Affiliations:** 1 Department of Orthopaedics, the Second Affiliated Hospital of Xuzhou Medical College, Xuzhou, JiangSu Province, China; 2 Department of Orthopedics, Affiliated Huai'an Hospital of Xuzhou Medical College, Huaian, JiangSu Province, China; University of South Alabama Mitchell Cancer Institute, UNITED STATES

## Abstract

Chemotherapeutic insensitivity remains a major obstacle to treating osteosarcoma effectively. Recently, increasing evidence has suggested that microRNAs (miRNAs) are involved in drug resistance. However, the effect of miR-138 on cisplatin chemoresistance in osteosarcoma has not been reported. We used real-time PCR to detect the expression of mature miR-138 in osteosarcoma tissues and cell lines. Cell proliferation, invasion, and migration assays were used to observe changes to the osteosarcoma malignant phenotype. MiR-138 was downregulated in osteosarcoma tissues and cell lines, and miR-138 overexpression negatively regulated osteosarcoma cell proliferation, migration, and invasion. We also verified that *EZH2* is a direct target of miR-138. Furthermore, enhancing *EZH2* expression reduced the inhibitory effects of miR-138 on osteosarcoma. Proliferation, apoptosis assays and caspase-3 activity assay confirmed that elevated miR-138 expression enhanced osteosarcoma cell chemosensitivity to cisplatin by targeting *EZH2*. In conclusion, the present study demonstrates that miR-138 acts as a tumor suppressor by enhancing osteosarcoma cell chemosensitivity and supports its potential application for treating osteosarcoma in the future.

## Introduction

With an estimated worldwide incidence rate of four per million per year, osteosarcoma is a major malignant bone and joint tumor with high morbidity in children and adolescents [1, 2]. With the recent advances in surgery, radiation therapy, and adjuvant chemotherapy, the 5-year survival rate of patients without metastasis at diagnosis is about 60–70% [3]. However, patients with metastatic or recurrent osteosarcoma have extremely poor survival outcomes [4]. Adjuvant chemotherapy, such as cisplatin, doxorubicin, and methotrexate, is frequently used for treating osteosarcoma [5]. However, chemotherapy-related problems, including severe side effects, persist. Presently, no efficient and established osteosarcoma biomarkers provide a basis for chemotherapy, and dose-limiting toxicity restricts the utility of drugs. Thus, it is urgent to identify new therapeutic targets and approaches for treating osteosarcoma.

MicroRNAs (MiRNAs), which are small, noncoding RNA molecules that inhibit specific target mRNAs by binding their 3′ untranslated regions (UTR), play important roles in oncogenesis [6–8]. MiRNAs are differentially expressed in various tissues and cells, and it has been suggested that they act as biomarkers or therapeutic targets, especially as oncogenes or tumor suppressors, by regulating their target genes [9]. To date, the role of miRNAs in osteosarcoma development and metastasis is unclear. Namløs et al. [10] reported that 177 miRNAs were differentially expressed in osteosarcoma cell lines, outlining the complex genetic project of elucidating osteosarcoma development and progression. Therefore, identifying specific miRNAs in osteosarcoma might provide potential therapeutic targets for treating osteosarcoma.

In the present study, we investigated the role of miR-138 in osteosarcoma progression. First, we confirmed that miR-138 was downregulated in osteosarcoma tissues and cell lines. Second, we detected osteosarcoma cell proliferation, migration, and invasion following miR-138 overexpression in osteosarcoma cell lines. Third, we found that *EZH2* was a direct target of miR-138 and that forced *EZH2* expression restored the inhibitory effects of miR-138 in osteosarcoma. Finally, we demonstrate that miR-138 enhances osteosarcoma cell chemosensitivity to cisplatin by targeting *EZH2*. In conclusion, our data suggest the important role of miR-138 in the osteosarcoma process and indicate its potential application in osteosarcoma therapy.

## Materials and Methods

### Human tissue samples

Pathology-confirmed, pre-chemotherapy osteosarcoma tissue samples (n = 20) and adjacent non-tumor tissues (n = 20) were acquired from patients who were undergoing surgery at the Affiliated Huai’an Hospital of Xuzhou Medical College Department of Orthopedics (osteosarcoma tissue samples, n = 13; adjacent non-tumor tissues, n = 15) and the Second Affiliated Hospital of Xuzhou Medical College Department of Orthopaedics (osteosarcoma tissue samples, n = 7; adjacent non-tumor tissues, n = 5) between February 2010 and February 2014. All tissues were obtained at surgery and immediately snap-frozen in liquid nitrogen. Written informed consent was obtained from all patients (for pediatric patients, on-behalf consent was obtained from their parents) who agreed to participate in the study. The percentage of malignant cells in each osteosarcoma sample is more than eighty, and the proportion of malignant cells in adjacent normal tissues is less than 10 percent or none. The Ethics Review Committee of Xuzhou Medical College approved this study.

### Cell lines and cell culture

The normal osteoblast cell line NHOst, hFOB1.19 and human osteosarcoma cell lines (HOS, Saos-2, MG-63, U2OS) were purchased from the Chinese Academy of Medical Sciences and cultured in Dulbecco’s modified Eagle’s medium (DMEM, USA) supplemented with 10% fetal bovine serum (FBS, USA), penicillin (100 U/ml), and streptomycin (100 μg/ml). NHOst cells were cultured in osteoblast growth medium (USA). All cells were incubated at 37°C in 5% CO_2_.

### Cell transfection

Cells were seeded into 6-, 12-, 24-, or 96-well plates and incubated at 37°C and 5% CO_2_ overnight. MiR-138 mimic and its scramble mimic negative control (miR-NC) were synthesized by GenePharma (Shanghai, China). The cells were transfected with 20 nmol/L miR-138 mimic or miR-NC using Lipofectamine 2000 (Invitrogen, Carlsbad, CA, USA) according to the manufacturer’s protocol.

### RNA extraction and real-time PCR

Total RNA was extracted from tissues or cultured cells using TRIzol (Invitrogen) and a miRNeasy Mini Kit (Qiagen) according to the manufacturers’ instructions. TaqMan MicroRNA Assays (Invitrogen) were used for real-time PCR to qualify miRNAs [11]. RNA was converted to complementary DNA, and PCR was performed using SYBR Premix DimerEraser (Takara, Dalian, China) on a 7900HT system. The PCR conditions were as follows: 95°C for 15 s; 40 cycles at 95°C for 5 s; 55°C for 34 s; 72°C for 30 s; melting curve. The level of mature miR-138 was normalized relative to the U6 endogenous control, and fold changes were calculated using the comparative threshold cycle value (2^−ΔΔCT^) method. All of the above experiments were performed in triplicate.

### Cell proliferation assay

Cells were seeded into 96-well plates at 2000 cells per well and cultured for 1, 2, 3, 4, and 5 days after transfection. Cell Counting Kit-8 (CCK-8; China) was used to determine cell proliferation. Cells were incubated in 10% CCK-8 reagent at 37°C until visual color conversion occurred. Subsequently, the absorbance at 450 nm was measured using a microplate reader (USA). All experiments were carried out in triplicate.

### Cell invasion and migration assays

Cells transfected with miR-138 mimic and miR-NC were harvested 48 h after transfection for the cell invasion and migration assays. We used 24-well BD Matrigel invasion chambers (BD Biosciences, Cowley, UK) in accordance with the manufacturer’s instructions. Cells in serum-free DMEM were seeded into the top 8.0-mm pore membrane chamber coated with 30 mg/cm^2^ Matrigel per well; DMEM supplemented with 10% FBS was used to stimulate cell migration and invasion to the bottom chamber. After 24 h, cells remaining on the top side of the membrane were removed with a cotton swab; cells in the bottom chamber were stained with 0.1% crystal violet (Sigma-Aldrich, St. Louis MO, USA). Cells were counted at ×10 magnification. The migration assay was performed in the same manner as the invasion assay but without the Matrigel.

### Apoptosis assay

To detect apoptosis, cells underwent flow cytometry analysis using an Annexin V-FITC Apoptosis Detection Kit (Beyotime, China). Cells were stained with 5 μl annexin V–fluorescein isothiocyanate (FITC), 10 μl propidium iodide, and 400 μl 1× binding buffer for 15 min, and then analyzed by flow cytometry (FACSCanto II, BD Biosciences).

### Western blotting

For western blotting, cell lysates were prepared using radioimmunoprecipitation assay buffer (Beyotime); protein concentrations were measured using the bicinchoninic acid assay [12]. Proteins were separated electrophoretically using 10% sodium dodecyl sulfate–polyacrylamide gel electrophoresis and transferred to a polyvinylidene difluoride membrane. The membrane was inoculated with primary antibodies against EZH2 (Bioword, USA) and glyceraldehyde-3-phosphate dehydrogenase (GAPDH; Bioword, USA) at 4°C overnight, followed by secondary antibody (Pierce, Rockford, IL, USA) inoculation. Results were detected using an enhanced chemiluminescence system (Tanon, Shanghai, China).

### Luciferase assay

A fragment of the human *EZH2* 3′ UTR containing the miR-138 binding site was PCR-amplified and cloned downstream of the firefly luciferase gene in the pMIR-REPORT vector (Ambion, Austin, TX, USA). We replaced the miR-138 binding site seed sequence (CACCAGC) with CAGGACC, and the mutation fragment was cloned into the pMIR-REPORT vector to produce the pMIR-EZH2mut-3′-UTR vector. Cells were seeded into 24-well plates the day before transfection, and then cotransfected with the constructs and miR-138 mimic or miR-NC. Luciferase activity was measured after 48 h using the Dual Luciferase Reporter Assay System (Promega, Madison, WI, USA).

### Chemosensitivity array

Cisplatin (final concentration, 1.25–80 μM; Sigma-Aldrich) was added to cells that had been incubated in 96-well plates overnight. After 48 h, cell viability was assayed using CCK-8.

### Caspase-3 Activity Assay

The activity of caspase-3 was determined using the Beyotime caspase-3 activity kit. Cell lysates were prepared and incubated with reaction buffer containing caspase-3 substrate (Ac-DEVD- pNA) after the treatment as indicated. Assays were performed on 96-well plates by incubating 10 mL protein of cell lysate per sample in 80 mL reaction buffer containing 10 mL caspase-3 sub- strate (Ac-DEVD-pNA; 2 mM) at 378C for 2 h according to the manufacturer’s protocol. The reaction was then measured at 405 nm for absorbance.

### Statistical analysis

GraphPad Prism 5 (La Jolla, CA, USA) was used for all data analyses. Spearman’s rank test was used to assess the association between miR-138 expression levels and EZH2. Student’s *t*-test was used to determine the differences between groups. A p-value of <0.05 was considered statistically significant.

## Results

### MiR-138 was Downregulated in Osteosarcoma Tissues and Cell Lines

**Fig 1:**
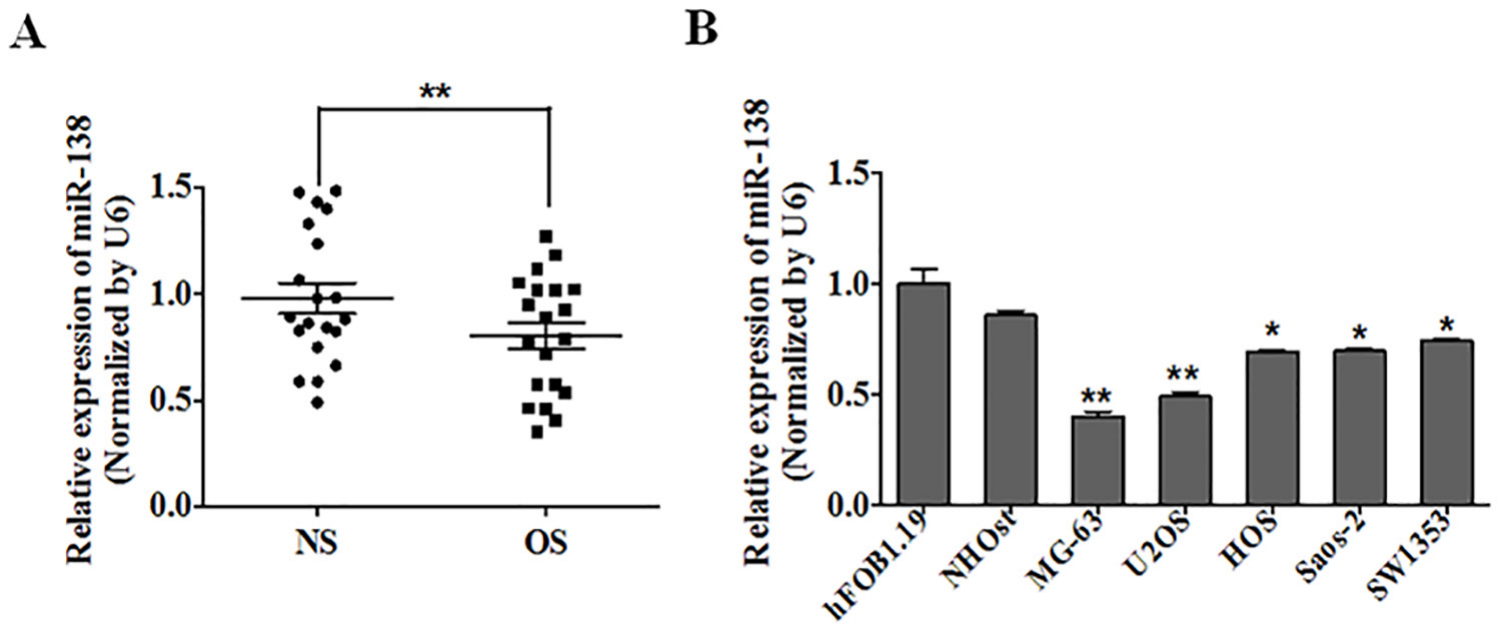
MiR-138 was frequently downregulated in osteosarcoma tissues and cell lines. (A) The expression of miR-138 was analyzed by qRT-PCR in 20 pairs of osteosarcoma samples (OS) and adjacent normal samples (NS). U6 was used as an internal control. (B) Relative miR-138 expression was determined in normal osteoblast cells (NHOst and hFOB1.19) and four osteosarcoma cell lines, HOS, Saos-2, MG-63 and U2OS.

To investigate miR-138 expression in osteosarcoma, we performed quantitative reverse transcription–PCR (qRT-PCR) on 20 paired samples of osteosarcoma and adjacent normal tissues. We found that miR-138 expression levels were significantly lower in the osteosarcoma tissues as compared with the normal tissues (P<0.01) (Fig 1A). MiR-138 was also downregulated in osteosarcoma cells. MiR-138 expression in the HOS, Saos-2, MG-63, and U2OS cell lines was significantly decreased as compared with the normal NHOst and hFOB1.19 osteoblast cells (Fig 1B)(Table 1).

**Table 1:** Expression profiles of miR-138 in Osteosarcoma Cells.

qRT-PCR		
	p-value	Fold change
NHOst vs hFOB1.19	0.1118	0.86
MG-63 vs hFOB1.19	0.0010	0.40
U2OS vs hFOB1.19	0.0019	0.49
HOS vs hFOB1.19	0.0103	0.69
Saos-2 vs hFOB1.19	0.0107	0.70
SW1353 vs hFOB1.19	0.0194	0.75

### MiR-138 Negatively Regulated Osteosarcoma Cell Proliferation, Migration, and Invasion

**Fig 2:**
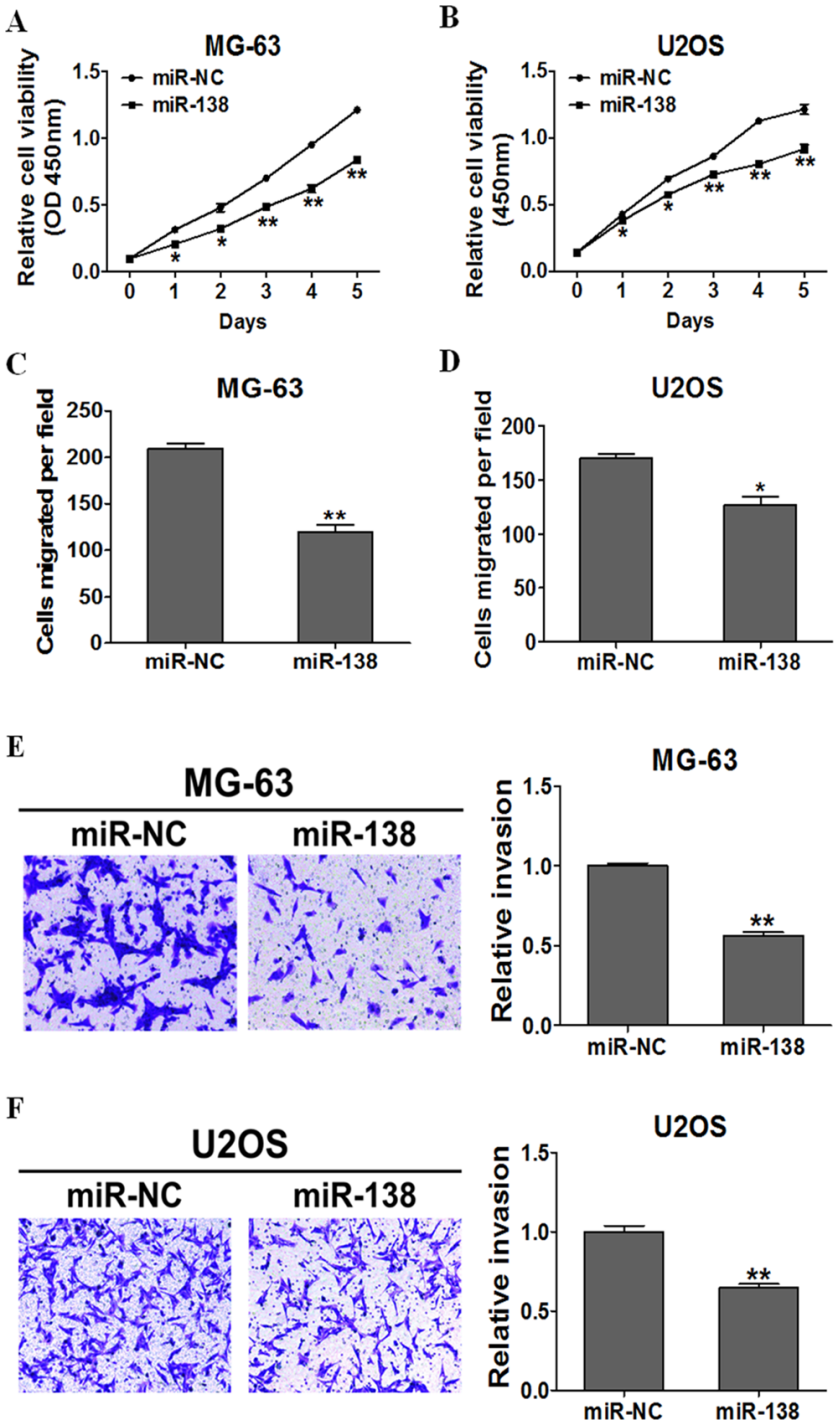
miR-138 negatively regulates osteosarcoma cell proliferation, migration and invasion. (A) (B)The Cell Counting Kit-8 (CCK-8) assay showed that MG-63 and U2OS cells stably expressing miR-138 grew slower than cells stably expressing miR-NC., (C) (D) (E) (F) Transwell migration and invasion assays of MG-63 and U2OS cells stably expressing miR-NC or miR-138 were performed.

The exact role of miR-138 in osteosarcoma metastasis remains to be clarified. To address this question, we transfected MG-63 and U2OS cells with miR-138 mimic or miR-NC and evaluated their proliferation, migration, and invasion ability. The CCK-8 proliferation assay showed that the cell growth rate was reduced in miR-138 mimic–transfected MG-63 and U2OS cells as compared with miR-NC–transfected cells. The MG-63 cells promotion efficiencies were 34.3%, 32.9%, 30.6%, 34.5% and 30.9%, respectively, and the U2OS cells promotion efficiencies were 11.1%, 17.1%, 15.8%, 28.9% and 24.4%, respectively. (Fig 2A and 2B). We used the migration and invasion assays to investigate the effects of miR-138 on osteosarcoma. Cells treated with miR-138 mimic were distinctively less migratory and invasive in comparison to the controls (Fig 2C–2F).

### *EZH2* was a Direct Target of miR-138 and Forced *EZH2* Expression Restored the Inhibitory Effects of miR-138

**Fig 3:**
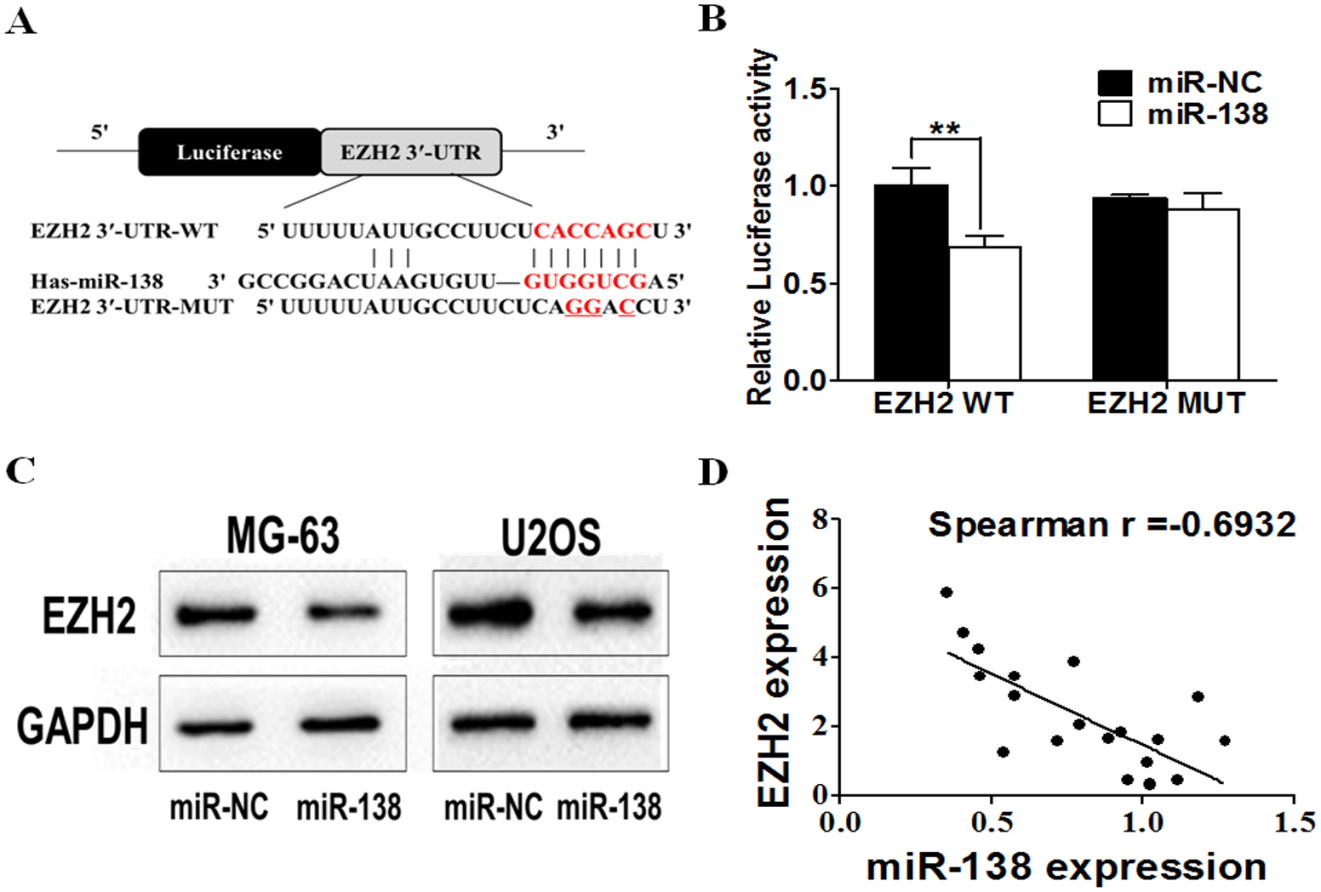
EZH2 is a direct target of miR-138 in osteosarcoma cells. (A) Sequence of the miR-138-binding site within the human EZH2 3′-UTR and the mutated EZH2 3′-UTR sequence. (B) Luciferase assay on MG-63 cells, which were co-transfected with miR-NC or miR-138 and a luciferase reporter containing the full length of EZH2 3′-UTR(WT) or a mutant(Mut). Luciferase activities were measured 24 hours post transfection. (C) The expression levels of EZH2 were decreased in cells with miR-138 overexpression by western blotting (D) Spearman′s correlation analysis was used to determine the correlation between the expression levels of EZH2 and miR-138 in 20 pairs of adjacent normal and osteosarcoma samples.

To elucidate the underlying mechanisms of miR-138 in osteosarcoma, we searched for candidate target genes of miR-138 using three mainstream target prediction databases: TargetScan (http://www.targetscan.org/), miRanda (http://www.microrna.org/microrna/home.do), and PicTar(http://pictar.mdc-berlin.de/). A conserved domain within the 3′ UTR of *EZH2* with a potential miR-138 binding site was identified (Fig 3A). Luciferase assay was performed on MG-63 cells to confirm this prediction. MG-63 cells were cotransfected with the wild-type (WT) or mutated (Mut) EZH2 luciferase reporter vector together with miR-138 mimic or miR-NC. MiR-138 overexpression significantly reduced WT reporter luciferase activity, but not that of the Mut reporter (Fig 3B), indicating that miR-138 directly targets the *EZH2* 3′ UTR. We conducted western blotting to confirm this finding: Fig 3C shows that miR-138 overexpression markedly reduced EZH2 protein levels (0.32-fold change in MG-63 cells, 0.55-fold change in U2OS cells). Spearman’s correlation analysis was used to determine the correlation between *EZH2* and miR-138 expression levels in clinical tissues, and revealed that miR-138 expression is negatively correlated with *EZH2* expression (Spearman r = -0.6932)(p = -0.0007). These results are further proof of the relationship between miR-138 and *EZH2*.

**Fig 4:**
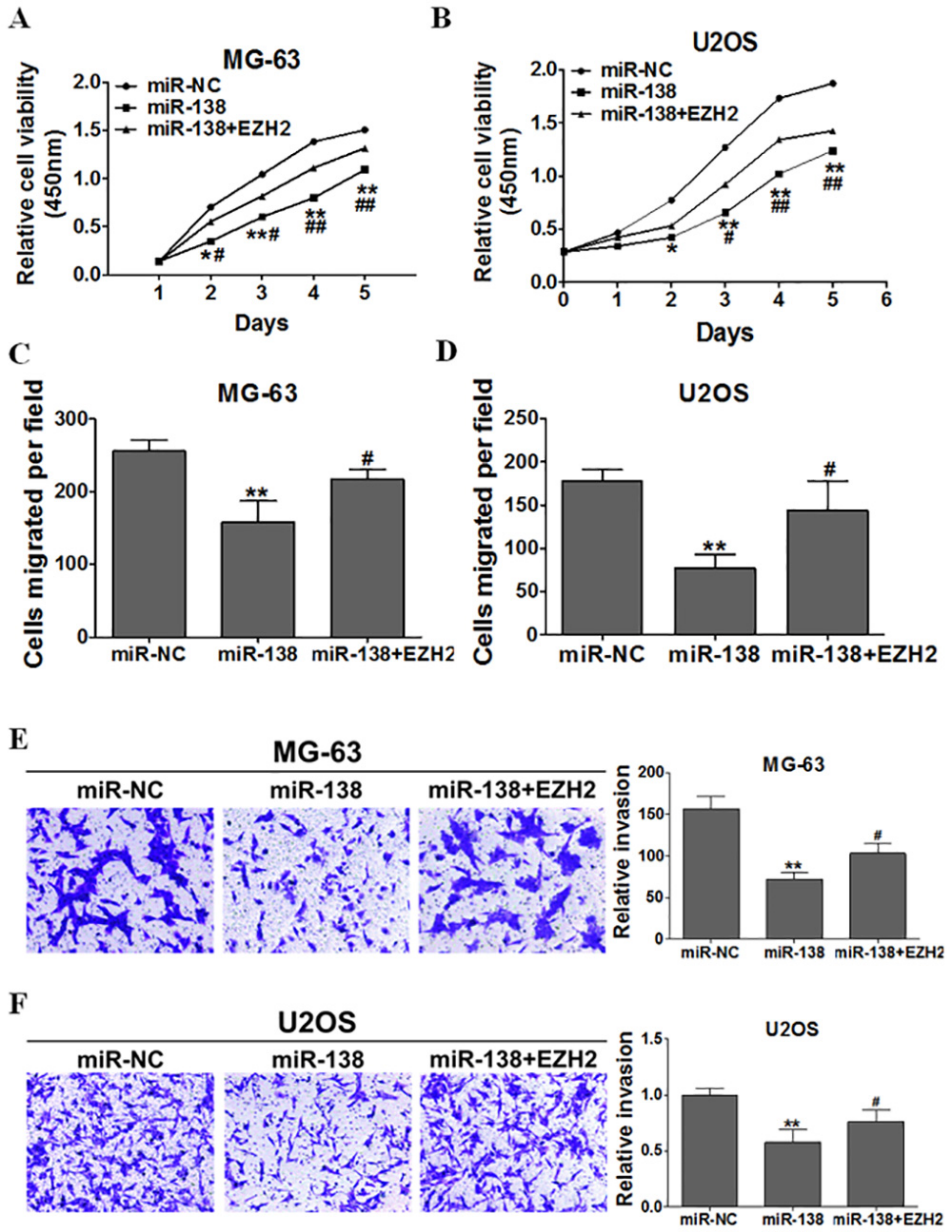
Forced expression of EZH2 restored inhibitory effects of miR-138. Overexpression of miR-138 arrested cell proliferation(A)(B), migration(C)(D), and invasion(E)(F),but these were rescued upon coexpression of exogenous EZH2 in MG-63 and U2OS cells.

Rescue experiments were performed to further validate that *EZH2*, as a target gene, is involved in the miR-138–induced anti-tumor process in osteosarcoma cells. MG-63 and U2OS cells were transfected with miR-NC, miR-138 mimic, or both miR-138 and *EZH2* expression vectors. EZH2 significantly attenuated the inhibition of osteosarcoma cell proliferation, migration, and invasion induced by miR-138 overexpression (Fig 4A–4F).

### Elevated Expression of miR-138 Enhanced Osteosarcoma Cell Chemosensitivity to Cisplatin by Targeting *EZH2*

**Fig 5:**
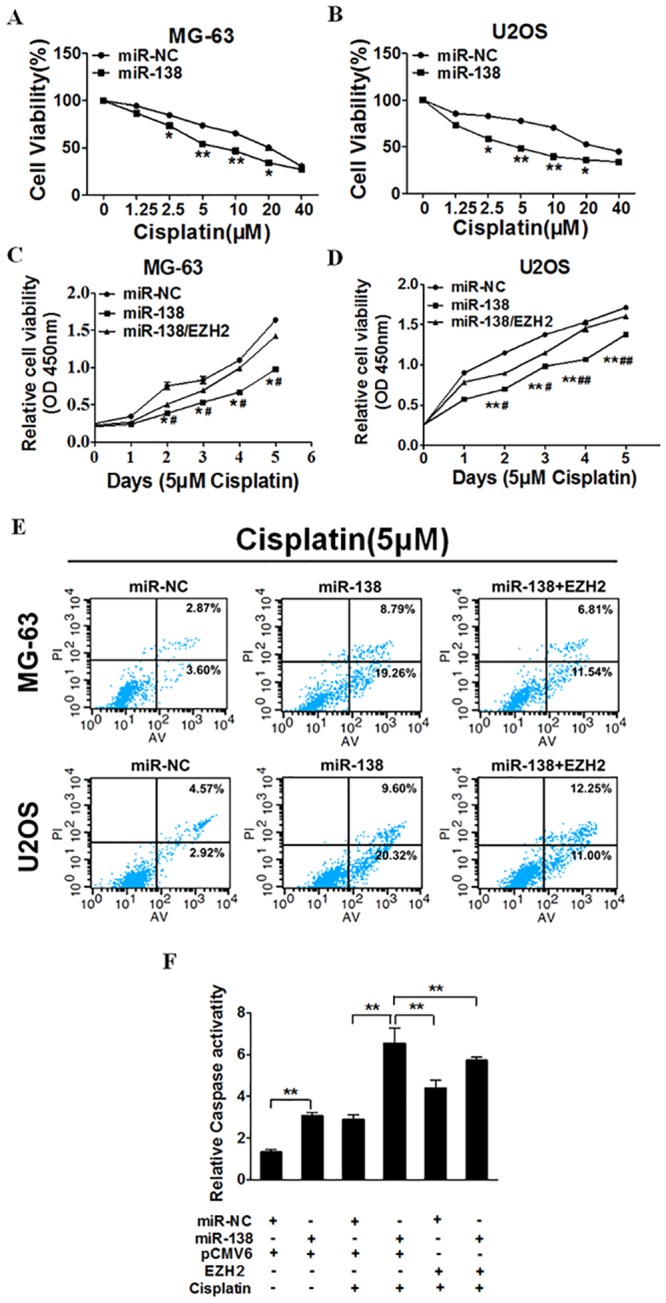
Elevated expression of miR-138 enhance the chemosensitivity of osteosarcoma cells to cisplatin by its target EZH2. (A) (B) MG-63 stably expressing miR-NC or miR-138 were pretreated with various concentration of cisplatin for 48h, and subjected to CCK-8 Assay. (C) (D) MG-63 and U2OS stably expressing miR-NC, miR-138 or miR-138 forced expression of EZH2 were pretreated with 5 μM of cisplatin for definite time points, and subjected to CCK-8 Assay, apoptosis analysis by flow cytometry(E). MG-63 cells stably expressing miR-NC or miR-138 were transfected with 2 mg pCMV6 vector, pCMV6 –EZH2 cDNA plasmid and cultured with or without cisplatin. After 72 h, the relative caspase-3 activities were determined. ** indicates significant difference at P <0.01(F).

Chemoresistance is one of the major factors of chemotherapy failure. To explore the potential role of miR-138 in osteosarcoma, MG-63 and U2OS cells were stably transfected with miR-138 mimic and miR-NC and exposed to cisplatin. MiR-138 overexpression significantly increased osteosarcoma cell chemosensitivity to cisplatin (Fig 5A and 5B). Furthermore, we restored *EZH2* expression by transfecting EZH2 expression vectors into MG-63 and U2OS cells with miR-NC or miR-138 mimic, and performed proliferation and apoptosis assays. *EZH2* overexpression partially abolished the effect induced by miR-138 plus cisplatin treatment (Fig 5C and 5D), Moreover, we found that the activity of caspase-3, a key executor of cell apoptosis, was significantly up-regulated upon treatment by miR-138 + cisplatin compared with miR-138 or cisplatin treatment alone, whereas EZH2 overexpression attenuated the activation of caspase-3 induced by miR-138 + cisplatin treatment. These results indicate that combining miR-138 and cisplatin induce an inhibitory effect in osteosarcoma by targeting *EZH2*.

## Discussion

Cisplatin, which is widely used in chemotherapeutic therapy, is effective for treating patients with osteosarcoma [13], markedly increasing the survival rate. However, via various mechanisms, chemoresistance is one of the main obstacles preventing this increase [14]. It is well known that miRNA dysregulation influences tumor malignant progression. It has been demonstrated that miRNAs also play important roles in cancer chemoresistance [15, 16]. Consequently, our purpose was to elucidate the mechanism of cisplatin resistance and to discover possible means of improving osteosarcoma treatment.

In the present study, we first confirmed that miR-138 is downregulated in osteosarcoma tissues and cell lines. Different miRNA expression profiles in osteosarcoma have been identified previously [17–19]. MiR-138 plays important roles different cancers. For example, Ma et al. found that, compared to normal cells, miR-138 expression is frequently reduced in gallbladder carcinoma cells and that miR-138 overexpression inhibits cell proliferation by directly suppressing BAG-1 expression [20]; Long et al. reported that miR-138 downregulation in human colorectal cancer (CRC) may be a new CRC prognostic biomarker, as miR-138 suppresses CRC cell migration and invasion, at least in part, by inhibiting the oncogene *TWIST2* [21]. MiR-138 also acts as a potential tumor suppressor that inhibits cell proliferation by targeting *PDK1* in non–small cell lung cancer (NSCLC) cells [22]. In accordance with these previous results, we verified that miR-138 negatively regulates osteosarcoma cell proliferation, migration, and invasion, identifying a new stage for miRNA research in osteosarcoma. Consequently, we believe that miR-138 contributes to the development and regulation of cisplatin resistance in osteosarcoma. Our subsequent transfection experiments confirmed that miR-138 overexpression alters the degree of cisplatin resistance in osteosarcoma cells. However, the specific regulatory mechanism remains unclear.

MiRNA function primarily relies on the target gene(s). To explore the potential mechanism between miR-138 and cisplatin resistance, we performed bioinformatics analysis. Our data indicated that, in osteosarcoma cells, *EZH2* is a direct target of miR-138, where *EZH2* expression is negatively correlated with that of miR-138 in osteosarcoma. A member of the histone methyltransferase family on 7q36.1, EZH2 catalyzes the trimethylation of histone H3 at lysine 27 (H3K27me3) [23]. It plays an important role in tumorigenesis through epigenetic gene silencing and chromatin remodeling [24]. *EZH2* overexpression was first reported in prostate and breast cancer [25, 26]. Subsequently, it was also reported in bladder cancer [27], SCLC and NSCLC [28], and brain tumors [29]. EZH2 overabundance in cancer cells may result from different mechanisms. MiR-25, -26a, -30d, -98, -101, -124, -137, -138, -144, -214, and let-7 interact with defined sequences within the *EZH2* 3′ UTR and directly downregulate EZH2 protein abundance [30]. Based on the above data, we performed bioinformatics analysis using the TargetScan, miRanda, and PicTar target prediction databases, identifying a potential miR-138 binding site in the 3′ UTR of *EZH2*. We used the luciferase assay and western blotting to confirm our findings.

As miR-138 influences the malignant phenotype of osteosarcoma, the suspicion of the influence of chemoresistance was proposed. In osteosarcoma tissues, few tumor suppressor/target chemoresistance axes have been demonstrated to participate in osteosarcoma tumorigenesis. In addition, several miRNAs are involved in osteosarcoma chemoresistance. For example, Xu et al. found that miR-34c inhibits osteosarcoma metastasis and chemoresistance [31]. Zhou et al. confirmed that miR-33a is upregulated in chemoresistant osteosarcoma and that it promotes osteosarcoma cell resistance to cisplatin by downregulating *TWIST* [32]. However, the miRNA/target chemoresistance axis is so complex that more miRNA/target axes in osteosarcoma require elucidation. As far as we know, the present study is the first to propose the miR-138/*EZH2* chemoresistance axis in osteosarcoma. We confirmed that miR-138 enhances osteosarcoma cell chemosensitivity by directly targeting *EZH2* and that *EZH2* overexpression reverses the miR-138–dependent chemosensitivity, which identifies a new direction for chemotherapy.

There were several limitations to this study. First, we focused solely on miR-138 regulation of *EZH2*, and future studies should explore the role of the up/downstream miR-138/*EZH2* signaling pathway to describe a better interpretation of the role of this pathway in osteosarcoma. Second, while we showed that miR-138/*EZH2* modulated osteosarcoma cell response to cisplatin, we did not specifically assess whether the enhanced chemosensitivity is mainly attributed to miR-138/*EZH2*, as miR-138 may target other genes as well. Third, our experiments were limited to *in vitro* experiments, which hardly reflect clinical practice. Future studies focusing on clinical application with more tissues samples are still needed.

In conclusion, this study demonstrates that miR-138 acts as a tumor suppressor in osteosarcoma, inhibiting cell proliferation, migration, and invasion by downregulating *EZH2* expression. Mir-138 overexpression also enhances osteosarcoma cell chemosensitivity to cisplatin by targeting *EZH2*. Thus, miR-138 could be a new therapeutic target for osteosarcoma treatment in the future.
